# A Novel *ATM* Pathogenic Variant in an Italian Woman with Gallbladder Cancer

**DOI:** 10.3390/genes12020313

**Published:** 2021-02-22

**Authors:** Elisa De Paolis, Andrea Urbani, Lisa Salvatore, Laura Foca, Giampaolo Tortora, Angelo Minucci, Paola Concolino

**Affiliations:** 1 Molecular and Genomic Diagnostics Unit, Fondazione Policlinico Universitario A. Gemelli IRCCS, 00168 Rome, Italy; elisa.depaolis@guest.policlinicogemelli.it (E.D.P.); laura.foca@guest.policlinicogemelli.it (L.F.); angelo.minucci@policlinicogemelli.it (A.M.); 2 Department of Basic Biotechnological Sciences, Intensivological and Perioperative Clinics, Università Cattolica del Sacro Cuore, Largo F. Vito 1, 00168 Rome, Italy; 3 Department of Medical Oncology, Fondazione Policlinico Universitario Agostino Gemelli IRCCS, 00168 Rome, Italy; lisa.salvatore@policlinicogemelli.it (L.S.); giampaolo.tortora@policlinicogemelli.it (G.T.); 4 Medical Oncology, Università Cattolica del Sacro Cuore, Largo F. Vito 1, 00168 Rome, Italy

**Keywords:** next-generation sequencing, *ATM* gene, gallbladder cancer, germline mutations

## Abstract

Gallbladder carcinoma (GBC) is one of the most aggressive malignancies with poor prognosis and a high fatality rate. The disease presents in advanced stages where the treatment is ineffective. Regarding GBC pathogenesis, as with other neoplasia, this tumor is a multifactorial disorder involving different causative factors such as environmental, microbial, metabolic, and molecular. Genetic alterations can be germline or somatic that involving proto-oncogenes, tumor suppressor genes, cell cycle genes, and growth factors. The ataxia telangiectasia mutated (*ATM*) gene, coding a serine/threonine kinase involved in the early stages of the homologous recombination (HR) mechanism, is one of the most altered genes in GBC. Here, we present the molecular characterization of a novel germline *ATM* large genomic rearrangement (LGR) identified by next-generation sequencing (NGS) analysis in an Italian woman diagnosed with metastatic GBC at the age of 55. The results underline the importance of expanding the NGS approach in gallbladder cancer in order to propose new molecular markers of predisposition and prognosis exploitable by novel targeted therapies that may improve the response of patients with *ATM*-deficient cancers.

## 1. Introduction

Gallbladder carcinoma (GBC) is one of the most common malignant tumors of the extrahepatic bile ducts with a poor prognosis and a high fatality rate. It is commonly diagnosed at an advanced stage and the 5 year survival is less than 5% [1,2]. The cumulative risk of gallbladder cancer, from birth to age 74, is 0.26% for women and 0.25% for men [3]. The incidence in the United States (US) is lower than that around the world, with a rate of 1.4 per 100,000 among women and 0.8 among men. Incidence rates are highest in Eastern Europe, East Asia, and Latin America [4,5,6]. Regarding the genetic basis of GBC, as with other neoplasia, this tumor is a multifactorial disorder involving multiple genetic alterations seen in several ethnicities [7,8]. Even if the genetic basis of the development of GBC is still scarce, many studies were performed to understand how certain types of genetic alterations act in GBC [9,10,11]. Recently, D’Afonseca et al. performed a study to identify the most mutated genes in GBC through data mining of public repositories [12]. The authors reported that the ataxia telangiectasia mutated (*ATM*) gene was one of the 14 most altered genes in GBC [12]. Data from The Cancer Genome Atlas (2018) have shown that the frequency of *ATM* tissue pathogenic variants (PVs) in GBC is approximately 6.25% in GBCs. Compared with the United States, the frequency of *ATM* in the Chinese population is significantly higher (8.3% vs. 1.9%, *p* = 0.03) [8]. The serine/threonine kinase ATM is a core component of the DNA damage repair (DDR) pathway, acting in response to double-strand breaks (DSBs). In particular, it is involved in the early stages of the homologous recombination (HR) mechanism that leads to cell-cycle arrest via *TP53* and to DNA repair via *BRCA1/2* activation [13]. Germline *ATM* heterozygosity occurs in about 1% of the population and appears to increase cancer susceptibility [14]. Studies of family members known to be heterozygous for *ATM* germline variants showed an approximate 2–3-fold increased risk of cancer, and a 5–9-fold increased risk of breast cancer in women [14,15]. In particular, the relative risk of breast cancer in those younger than age 50 was increased [16]. Other studies identified functional *ATM* germline variants associated with increased risk of lung cancer, thyroid cancer, and familial pancreatic ductal adenocarcinoma [17,18,19,20,21]. Lastly, somatic *ATM* PVs are commonly found in lymphoid malignancies, as well as a variety of solid tumors [22,23,24,25,26]. Such variants may result in chemotherapy resistance and adverse prognosis, but may also be exploited by novel targeted therapies that may improve the response of patients with *ATM*-deficient cancers [27].

Here, we present the case of a patient diagnosed with GBC harboring a novel germline *ATM*-inactivating large genomic rearrangement (LGR). Familiar analysis and molecular characterization of the rearrangement were performed.

## 2. Materials and Methods

### 2.1. Patients

A woman from southern Italy received a diagnosis of GBC and peritoneal and ovarian metastases at the age of 55. In July 2020, she performed the following diagnostic exams: a total-body computed tomography (CT) scan (with evidence of pelvic mass, peritoneal metastases, and gallbladder with a thickened wall), a transvaginal ultrasound (with evidence of a right ovarian solid multilocular lesion of 225 × 125 × 243 mm and a left ovarian solid multilocular lesion of 128 × 128 × 124 mm), and tumoral marker dosage (Carcino-Embryonic Antigen (CEA): 8.19 ng/mL (normal range < 5.0 ng/mL), Carbohydrate Antigen (CA) 19.9: 401.90 U/mL (normal range < 37.0 UI/mL), and CA-125: 887.00 U/mL (normal range < 35.0 UI/mL)). The patient underwent surgery (laparotomy with total hysterectomy with Douglas peritonectomy and prevesical peritoneum, bilateral annexiectomy, radical omentectomy, splenectomy, bowel loop nodule removal, and right diaphragmatic peritoneum nodule removal) for suspected ovarian cancer on August 2020. The histological evaluations revealed a moderately differentiated (G2) gallbladder adenocarcinoma with ovarian and peritoneal metastasis. In September 2020, postoperative total-body CT scan resulted negative for macroscopic disease and tumoral markers were as follows: CEA, 0.6 ng/mL; CA 19.9, 32.5 U/mL; CA 125, 34 U/mL. In October 2020 the patient started “adjuvant” treatment with cisplatin plus gemcitabine, which is still ongoing. 

**Figure 1:**
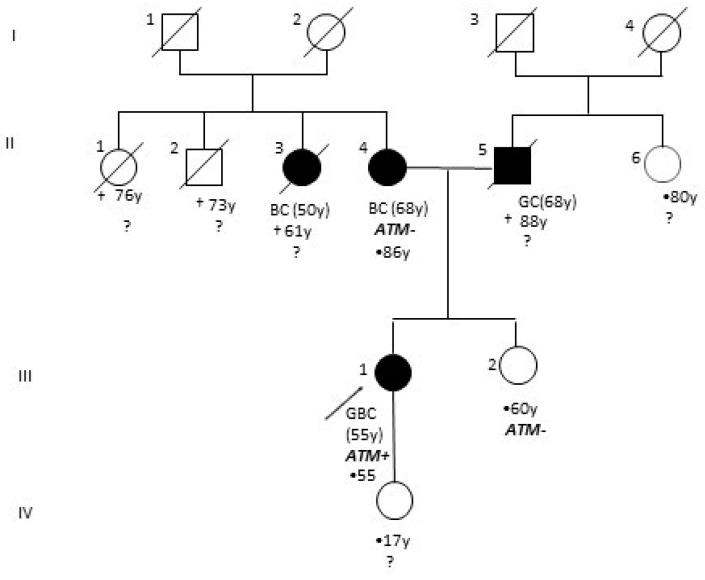
Patient’s family pedigree with four generations depicted. Black circles and squares indicate individuals affected by cancer. The proband is indicated with an arrow. GC: gastric cancer, BC: breast cancer, GBC: gallbladder cancer, y: years at diagnosis of cancer, †: age of death, •: current age, ataxia telangiectasia mutated (ATM)+: *ATM* mutated, ATM−: negative on *ATM* screening, ?: untested for *ATM* mutations.

The woman presented a positive family history of cancer; her mother had a monolateral breast cancer at the age of 68 and her father was affected by gastric cancer (Figure 1). She was referred from the Service of Medical Oncology to our molecular diagnostic unit. Informed consent and a blood sample were obtained to allow genetic analysis of 26 cancer-related genes. As an *ATM* LGR was identified, close relatives, i.e., the sister and the proband’s mother, were invited to provide informed consent and undergo genetic testing.

### 2.2. Next-Generation Sequencing (NGS) and Large Genomic Rearrangement (LGR) Detection

Genomic DNA was isolated from peripheral blood samples using a Maxwell 16 Blood DNA Purification kit (Promega, Madison, WI, USA) and Maxwell 16 MDx AS3000 instrument (Promega). Massive parallel sequencing (MPS) was carried out using the Hereditary Cancer Solution (HCS) Kit (SOPHIA GENETICS, Saint-Sulpice, Switzerland) on an Illumina MiSeq instrument (Illumina, San Diego, CA, USA). Sequencing data were analyzed via Sophia DDM^®^ software v.4.2. (SOPHIA GENETICS). The HCS kit performs the analysis of 26 cancer-related genes (*ATM*, *APC*, *BARD1*, *BRCA1*, *BRCA2*, *BRIP1*, *CDH1*, *CHEK2*, *EPCAM*, *FAM175A*, *MLH1*, *MRE11A*, *MSH2*, *MSH6*, *MUTYH*, *NBN*, *PALB2*, *PIK3CA*, *PMS2*, *PMS2C*, *PTEN*, *RAD50*, *RAD51C*, *RAD51D*, *STK11*, *TP53*, and *XRCC2*).

A Multiplex Ligation-Dependent Probe Amplification (MLPA) assay was performed, as a further method, when the new rearrangement was detected. The SALSA MLPA kit for *ATM* (P041 and P042; MRC Holland, Amsterdam, The Netherlands) was used according to the manufacturer’s instructions. Amplicons were run on an ABI 3500 Genetic Analyzer (Thermo Fisher Scientific, Foster City, CA, USA), and the collected data were analyzed using Coffalyser.NET Software (MRC Holland). Three healthy males and three healthy females were included in the analysis as wild-type controls.

### 2.3. Analysis of Breakpoint Region

To characterize the breakpoint region, deletion-specific PCR primers (Del18F 5′–TGTGTGTAACTACTGCTCAG–3′ and Del28R 5′–TGCTTTAATCACATGCGATGG–3′) producing a PCR product of 2620 bp were designed. PCR reactions were performed using a long-range PCR kit (Expand Long Template PCR System, Roche Applied Science, Monza, Italy). The Del28R primer was used in sequencing analysis. The PCR product was sequenced using a BigDye Terminator Cycle Sequencing Kit v3.1 (Thermo Fisher Scientific) and an ABI 3500 Genetic Analyzer (Thermo Fisher Scientific). Results were analyzed with the SeqScape v2.5 software package (Thermo Fisher Scientific) using NG_009830.1 as a reference.

### 2.4. RT-PCR

Total RNA was isolated from peripheral blood lymphocytes with TRIzol reagent (Thermo Fisher Scientific). Synthesis of complementary DNA (cDNA) was performed with SuperScript II Reverse Transcriptase (Thermo Fisher Scientific) using DNAase-treated RNA in the presence of random primers and RNAaseOUT (Thermo Fisher Scientific). cDNA amplification was performed using the following primers: R18del 5′–GCCATTAATCCTTTAGCTGA–3′ and R28del 5′–GGTTTTATGACAATTGCTG–3′. A PCR fragment of 288 bp, harboring the deletion, was extracted from agarose gel (QIAquick Gel Extraction Kit, Qiagen Hilden, Germany) and sequenced using the same couple of primers.

## 3. Results

### 3.1. NGS Analysis and LRG Detection

**Figure 2:**
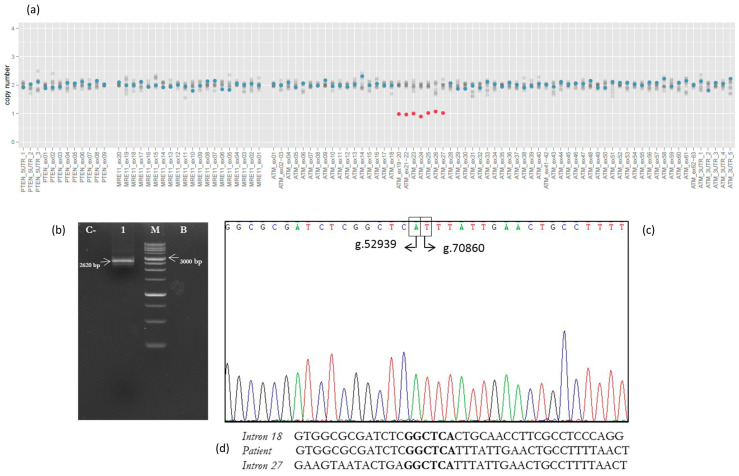
Characterization of exon 19–27 deletion in the *ATM* gene. (**a**) Copy number variation (CNV) analysis by Sophia DDM® software v.4.2. Amplicon coverage: *ATM* amplicons related to exons 19–27 show a copy number (CN) value of 1 (normal value: CN = 2). (**b**) Genomic DNA was amplified using specific deletion primers (Del18F and Del28R). The mutant allele gives rise to a 2620 bp fragment. C−: negative control (wild-type DNA), Lane 1: patient, M: marker, B: blank. (**c**) The electropherogram of the 2620 bp PCR fragment, containing the deletion’s breakpoint, showed a wild-type sequence until the nucleotide g.52939A (NG_009830.1) of *ATM* intron 18. The following sequence corresponded to the *ATM* intron 27 starting from the g.70860T nucleotide. (**d**) The very short homologous sequence of six nucleotides (GGCTCA) identified at the breakpoint region.

No small insertions/deletions or point mutations were detected in the 26 cancer-related genes investigated by the HCS NGS Kit. However, NGS copy number variation (CNV) prediction analysis identified a large *ATM* deletion, involving exons 19–27, in our patient (Figure 2a). This result was confirmed by performing the MLPA assay on a fresh DNA sample. Successively, the proband’s parents, screened by *ATM* MLPA analysis, resulted negative (data available on request).

### 3.2. Analysis of Breakpoint Region

The PCR fragment of 2620 bp, containing the breakpoint region, showed a wild-type sequence until the nucleotide g.52939A (NG_009830.1) of *ATM* gene intron 18. The following sequence corresponded to the *ATM* intron 27 starting from the g.70860T nucleotide (Figure 2b,c). We report the novel *ATM* rearrangement in agreement with the recommended HGVS nomenclature as NG_009830.1:g.52939_70860del.

### 3.3. RT-PCR Results

**Figure 3:**
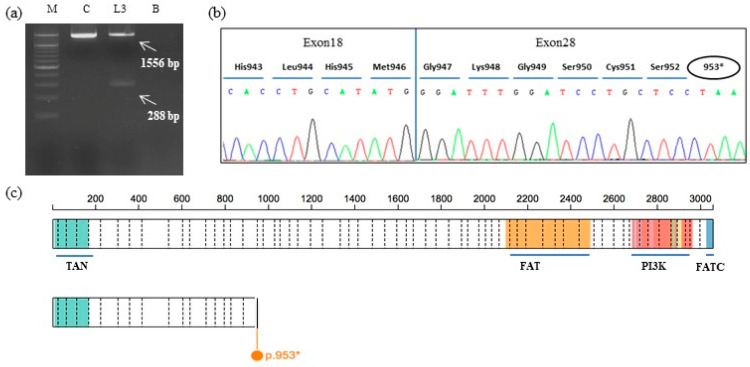
Results from messenger RNA (mRNA) analysis. (**a**) A single PCR fragment of 1556 bp was obtained from the complementary DNA (cDNA) of the wild-type control, while two fragments of 1556 and 288 bp were amplified using the cDNA of the patient. M: marker, C: cDNA wild-type control, Lane 3: patient; B: blank. (**b**) Electropherogram showing the sequence of the PCR product of 288 bp, containing the expected deletion. Sequencing analysis revealed a wild-type sequence until the nucleotide c.3223G (NM_000051.3) of the *ATM* gene in exon 18 (Met946). The following sequence corresponded to *ATM* exon 28 starting from the nucleotide c.4495G (NM_000051.3) (Gly947). A stop codon at amino acid 953 (953*) produced a truncated protein. (**c**) The figure shows the schematic maps of the full-length ATM protein (3056 amino acids; top) and the predicted truncated ATM protein (953 amino acids; bottom). Main protein domains are represented by colored areas and described, whereas the vertical lines represent exon boundaries (starting from exon 2). TAN: Tel1/ATM N-terminal motif; FAT: Frap–Atm–Trrap domain; PI3K: Phospho-Inositide 3-Kinase domain; FACT: Frap–Atm–Trrap Carboxy-Terminal domain (https://proteinpaint.stjude.org/).

A single PCR fragment of 1556 bp was obtained from the cDNA of the control, while two fragments of 1556 and 288 bp were amplified using cDNA of the patient (Figure 3a). A PCR product of 288 bp, containing the expected deletion, was cut out and isolated from agarose gel, sequenced with appropriate primers, and analyzed. Sequencing analysis revealed a wild-type sequence until the nucleotide c.3223G (NM_000051.3) of the *ATM* gene in exon 18. The following sequence corresponded to the *ATM* exon 28 starting from the nucleotide c.4495G (NM_000051.3) (Figure 3b). The *ATM* exon 19–27 deletion disrupted the reading frame of the messenger RNA (mRNA), producing a premature stop codon and a truncated protein of 952 amino acids (NP_000042.3:p.(Tyr947GlyfsTer7)) (Figure 3b).

## 4. Discussion

We present the molecular characterization of a novel germline *ATM* LGR identified by NGS analysis in an Italian women diagnosed with GBC. As the family history revealed several cases of cancer in close relatives (Figure 1), a germline analysis of 26 cancer-related genes was requested by the oncologist. The SOPHIA GENETICS HCS gene panel identified the deletion of exons 19–27 in the *ATM* gene. This result was confirmed by MLPA analysis, and molecular studies, involving long-range PCR and Sanger sequencing, were performed in order to characterize the novel rearrangement. In the mutated allele, exon 28 is juxtaposed to exon 18, disrupting the mRNA reading frame and generating a truncated protein of 952 amino acids missing important functional domains [28]. Analysis of the breakpoint region did not show any sequence that could be involved in the rearrangement process, such as Alu sequences or recombination association motifs. However, a very short homologous sequence of six nucleotides (GGCTCA) was identified at the breakpoint site (Figure 2d).

Family analysis revealed that both the proband’s mother and the proband’s sister resulted negative on *ATM* screening. No information was available regarding the proband’s father, who was diagnosed with gastric cancer at the age of 68 and died 20 years later. Therefore, it remains unsolved if the novel *ATM* rearrangement is the result of an inherited defect or a de novo PV. In the future, the proband’s daughter, if consenting, will benefit from the genetic test.

Germline ATM pathogenic variants are rarely reported in patients with biliary tract cancers [29,30,31]. To the best of our knowledge, this is the first case where a germline LGR has been identified in a patient with GB cancer. Our report underlines the importance of expanding NGS studies in gallbladder cancer and strengthens the need to generate more knowledge regarding the most important alterations in this tumor, to propose new molecular markers of predisposition and prognosis. In particular, NGS results could be useful for such patients with no better treatment options in order to establish personalized treatment approaches. In fact, patients with homologous recombination deficiency (HRD) are considerably more likely to respond to drugs that impact DNA stability including platinum drugs and poly (ADP-ribose) polymerase (PARP) inhibitors [32], as recently reported by Zhang et al. [33]. These authors described the first case showing the clinical benefits of olaparib treatment in a patient with GBC harboring an *ATM*-inactivating mutation found in combination with a *STK11* frameshift variant. In this case, NGS analysis was performed on the tumor and no information regarding the germline origin of the two variants was provided [33]. The woman had a progression-free survival (PFS) of approximately 13 months following treatment with olaparib [33].

To date, our patient presents fair physical condition and she is undergoing first-line systemic therapy with gemcitabine-based chemotherapy. A CT scan performed 1 and 3 months after surgery did not show signs of disease progression. A second-line treatment with olaparib could be considered by oncologists.

## 5. Conclusions

The genetic basis of biliary tract cancer remains poorly understood and few data regarding the germline evaluation are available. Given the paucity of published data about the germline contribution of target genes in biliary tract cancer patients, sharing data about clinical case reports, molecular analysis of target cancer-predisposition genes and large genomic studies is needed. The molecular characterization could be beneficial for a genomic profiling-guided therapy.

## References

[1] Goetze T.O. (2015). Gallbladder carcinoma: Prognostic factors and therapeutic options. World J. Gastroenterol..

[2] Kanthan R., Senger J.-L., Ahmed S., Kanthan S.C. (2015). Gallbladder Cancer in the 21st Century. J. Oncol..

[3] Sung H., Ferlay J., Siegel R.L., Laversanne M., Soerjomataram I., Jemal A., Bray F. (2018). Global cancer statistics 2018: GLOBOCAN estimates of incidence and mortality worldwide for 36 cancers in 185 countries. CA Cancer J. Clin..

[4] Rawla P., Sunkara T., Thandra K.C., Barsouk A. (2019). Epidemiology of gallbladder cancer. Clin. Exp. Hepatol..

[5] Henley S.J., Weir H.K., Jim M.A., Watson M., Richardson L.C. (2015). Gallbladder Cancer Incidence and Mortality, United States 1999–2011. Cancer Epidemiol. Biomark. Prev..

[6] Torre L.A., Siegel R.L., Islami F., Bray F., Jemal A. (2018). Worldwide Burden of and Trends in Mortality from Gallbladder and Other Biliary Tract Cancers. Clin. Gastroenterol. Hepatol..

[7] Narayan R.R., Creasy J.M., Ms D.A.G., Gönen M., Kandoth C., Kundra R., Solit D.B., Askan G., Klimstra D.S., Basturk O. (2019). Regional differences in gallbladder cancer pathogenesis: Insights from a multi-institutional comparison of tumor mutations. Cancer.

[8] Yang P., Javle M., Pang F., Zhao W., Abdel-Wahab R., Chen X., Meric-Bernstam F., Chen H., Borad M.J., Liu Y. (2019). Somatic genetic aberrations in gallbladder cancer: Comparison between Chinese and US patients. HepatoBiliary Surg. Nutr..

[9] Sharma A., Kumar A., Kumari N., Krishnani N., Rastogi N. (2017). Mutational frequency of KRAS, NRAS, IDH2, PIK3CA, and EGFR in North Indian gallbladder cancer patients. Ecancermedicalscience.

[10] Quan Z.W., Wu K., Wang J., Shi W., Zhang Z., Merrell R.C. (2001). Association of p53, p16, and vascular endothelial growth factor protein expressions with the prognosis and metastasis of gallbladder cancer. J. Am. Coll. Surg..

[11] Kiguchi K., Carbajal S., Chan K., Beltrán L., Ruffino L., Shen J., Matsumoto T., Yoshimi N., DiGiovanni J. (2001). Constitutive expression of ErbB-2 in gallbladder epithelium results in development of adenocarcinoma. Cancer Res..

[12] D’Afonseca V., Arencibia A.D., Echeverría-Vega A., Cerpa L., Cayún J.P., Varela N.M., Salazar M., Quiñones L.A. (2020). Identification of Altered Genes in Gallbladder Cancer as Potential Driver Mutations for Diagnostic and Prognostic Purposes: A Computational Approach. Cancer Inform..

[13] Perkhofer L., Gout J., Roger E., De Almeida F.K., Simões C.B., Wiesmüller L., Seufferlein T., Kleger A. (2020). DNA damage repair as a target in pancreatic cancer: State-of-the-art and future perspectives. Gut.

[14] Swift M., Morrell D., Massey R.B., Chase C.L. (1991). Incidence of Cancer in 161 Families Affected by Ataxia–Telangiectasia. N. Engl. J. Med..

[15] Peterson R.D., Funkhouser J.D., Tuck-Muller C.M., Gatti R.A. (1992). Cancer susceptibility in ataxia-telangiectasia. Leukemia.

[16] Thompson D., Duedal S., Kirner J., McGuffog L., Last J., Reiman A., Byrd P., Taylor M., Easton D.F. (2005). Cancer Risks and Mortality in Heterozygous ATM Mutation Carriers. J. Natl. Cancer Inst..

[17] Liu J., Wang X., Ren Y., Li X., Zhang X., Zhou B. (2014). Effect of Single Nucleotide Polymorphism Rs189037 in ATM Gene on Risk of Lung Cancer in Chinese: A Case-Control Study. PLoS ONE.

[18] Shen L., Yin Z.-H., Wan Y., Zhang Y., Li K., Zhou B.-S. (2012). Association between ATM polymorphisms and cancer risk: A meta-analysis. Mol. Biol. Rep..

[19] Grant R.C., Al-Sukhni W., Borgida A.E., Holter S., Kanji Z.S., McPherson T., Whelan E., Serra S., Trinh Q.M., Peltekova V. (2013). Exome sequencing identifies nonsegregating nonsense ATM and PALB2 variants in familial pancreatic cancer. Hum. Genom..

[20] Roberts N.J., Jiao Y., Yu J., Kopelovich L., Petersen G.M., Bondy M.L., Gallinger S., Schwartz A.G., Syngal S., Cote M.L. (2011). ATM Mutations in Patients with Hereditary Pancreatic Cancer. Cancer Discov..

[21] Rustgi A.K. (2014). Familial pancreatic cancer: Genetic advances. Genes Dev..

[22] Wan Y., Wu C.J. (2013). SF3B1 mutations in chronic lymphocytic leukemia. Blood.

[23] Beltran H., Yelensky R., Frampton G.M., Park K., Downing S.R., MacDonald T.Y., Jarosz M., Lipson D., Tagawa S.T., Nanus D.M. (2013). Targeted Next-generation Sequencing of Advanced Prostate Cancer Identifies Potential Therapeutic Targets and Disease Heterogeneity. Eur. Urol..

[24] Biankin A.V., Initiative A.P.C.G., Waddell N., Kassahn K.S., Gingras M.-C., Muthuswamy L.B., Johns A.L., Miller D.K., Wilson P.J., Patch A.-M. (2012). Pancreatic cancer genomes reveal aberrations in axon guidance pathway genes. Nat. Cell Biol..

[25] Ding L., Getz G., Wheeler D.A., Mardis E.R., McLellan M.D., Cibulskis K., Sougnez C., Greulich H., Muzny D.M., Morgan M.B. (2008). Somatic mutations affect key pathways in lung adenocarcinoma. Nat. Cell Biol..

[26] Beggs A.D., Domingo E., McGregor M., Presz M., Johnstone E., Midgley R., Kerr D., Oukrif D., Novelli M., Abulafi M. (2012). Loss of expression of the double strand break repair protein ATM is associated with worse prognosis in colorectal cancer and loss of Ku70 expression is associated with CIN. Oncotarget.

[27] Choi M., Kipps T., Kurzrock R. (2016). ATM Mutations in Cancer: Therapeutic Implications. Mol. Cancer Ther..

[28] Xu B., Boohaker R.J. (2014). The versatile functions of ATM kinase. Biomed. J..

[29] Wardell C.P., Fujita M., Yamada T., Simbolo M., Fassan M., Karlic R., Polak P., Kim J., Hatanaka Y., Maejima K. (2018). Genomic characterization of biliary tract cancers identifies driver genes and predisposing mutations. J. Hepatol..

[30] Lin J., Dong K., Bai Y., Zhao S., Dong Y., Shi J., Shi W., Long J., Yang X., Wang D. (2019). Precision oncology for gallbladder cancer: Insights from genetic alterations and clinical practice. Ann. Transl. Med..

[31] Bs H.M., Stadler Z.K., Berger M.F., Solit D.B., Ly M., Lowery M.A., Mandelker D., Zhang L., Jordan E., El Dika I. (2020). Germline alterations in patients with biliary tract cancers: A spectrum of significant and previously underappreciated findings. Cancer.

[32] Pennington K.P., Walsh T., Harrell M.I., Lee M.K., Pennil C.C., Rendi M.H., Thornton A., Norquist B.M., Casadei S., Nord A.S. (2014). Germline and Somatic Mutations in Homologous Recombination Genes Predict Platinum Response and Survival in Ovarian, Fallopian Tube, and Peritoneal Carcinomas. Clin. Cancer Res..

[33] Zhang W., Shi J., Li R., Han Z., Li L., Li G., Yang B., Yin Q., Wang Y., Ke Y. (2020). Effectiveness of Olaparib Treatment in a Patient with Gallbladder Cancer with an ATM -Inactivating Mutation. Oncologist.

